# Reconstruction of an Irradiated Perineal Wound With a Superior Gluteal Artery Perforator Flap

**Published:** 2017-11-30

**Authors:** Yangshu L. Pan, Albert H. Chao

**Affiliations:** Department of Plastic Surgery, Ohio State University, Columbus

**Keywords:** perineal reconstruction, perforator, microsurgery, perforator flap, cervical cancer

## DESCRIPTION

**Figure 1:**
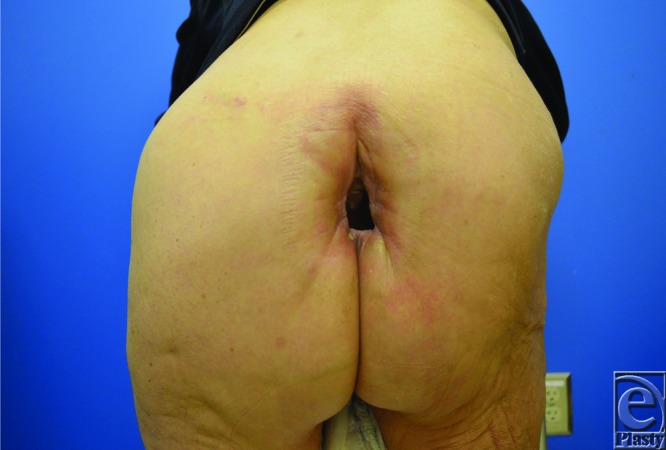
Perineal wound upon presentation in a patient with a history of cervical cancer after chemotherapy and radiation therapy. The defect had an internal diameter of approximately 6 cm, with significant dead space and exposed coccyx.

A 67-year-old woman presented with a nonhealing perineal wound following chemoradiation for cervical cancer. The defect measured 6 cm, with significant underlying dead space and exposed coccyx (Fig 1). The patient underwent reconstruction with a 2-perforator superior gluteal artery perforator (SGAP) flap to achieve wound closure while minimizing donor site morbidity.

## QUESTIONS

1. What flaps are traditionally used for perineal reconstruction?
2. What considerations guide flap selection in perineal reconstruction?
3. What are the advantages of the SGAP flap compared with traditional options?
4. What anatomic landmarks are important for designing a SGAP flap?

## DISCUSSION

Oncologic perineal defects can represent challenging reconstructive cases for the plastic surgeon. Contributory factors include location over a weight-bearing surface and site of shearing, and prior treatments such as radiation therapy. Traditional options for perineal reconstruction include both abdominally based and thigh-based flaps. The vertical rectus abdominis myocutaneous (VRAM) flap is the primary abdominally based flap that is utilized, and thigh-based flaps that are commonly used include the anterolateral thigh (ALT), gracilis, and posterior thigh flaps.

Flap selection depends on several factors, including defect size and location, quality of local tissues, and donor site morbidity. The VRAM flap provides a large amount of well-vascularized tissue including muscle and a skin paddle, although requires celiotomy and sacrifice of the rectus abdominis muscle and may not always be feasible such as in instances of fecal/urinary diversion or if the deep inferior epigastric vessels have been sacrificed. The gracilis flap can be harvested singularly or bilaterally depending on the situation and is associated with minimal donor site morbidity but has limited bulk and often requires skin grafting due to an inconsistently reliable skin paddle.^1^^,^^2^ The ALT flap provides a large amount of well-vascularized tissues including a skin paddle and the vastus lateralis muscle, if needed. Its potential downsides include possible need for donor site skin grafting if a large flap is necessary. Finally, the posterior thigh flap is a fasciocutaneous flap that is an option for perineal reconstruction. Its disadvantages include limited bulk and a relatively more difficult harvest from the supine position than from the prone position.

This patient presented with a posteriorly located irradiated perineal defect that exhibited significant depth and 3-dimensionality that necessitated dead space obliteration, which were all factors influencing flap selection. The defect location was largely beyond the arc of rotation of VRAM or ALT flaps. The need for dead space obliteration limited the use of other thigh-based flaps such as the gracilis and posterior thigh flaps. The SGAP flap was well positioned for a relatively posterior perineal defect and provided sufficient bulk for dead space obliteration. In addition, its blood supply was outside the zone of prior radiation therapy. Furthermore, the SGAP flap is a perforator flap that does not involve any muscle sacrifice and therefore entails minimal donor site morbidity. Finally, the SGAP flap is harvested from the prone position, obviating the need for any intraoperative position change for this posteriorly located perineal defect.

**Figure 2:**
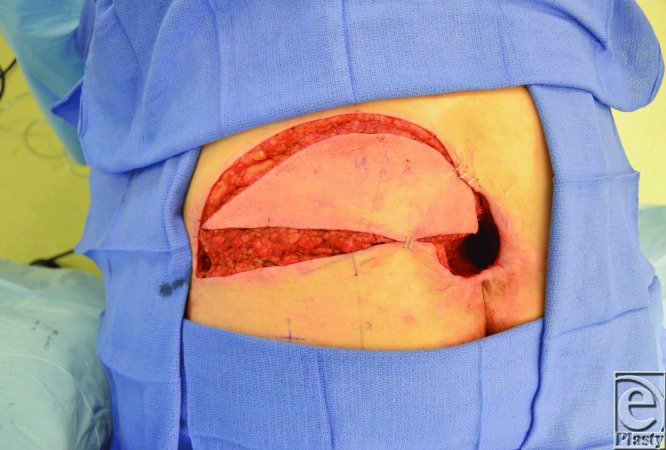
Skin paddle design of the left superior gluteal artery perforator flap.

**Figure 3:**
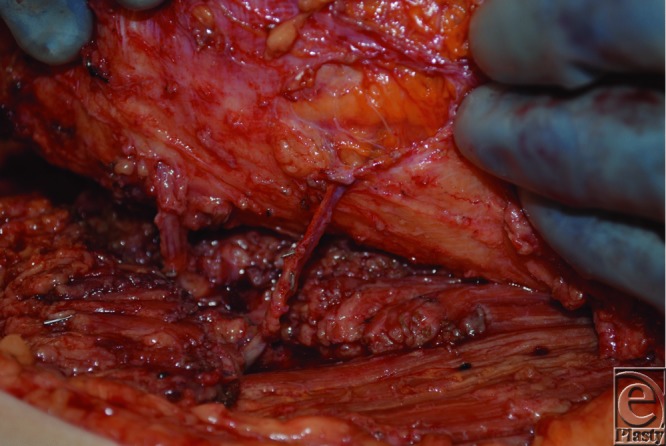
Two superior gluteal artery perforators were identified and centrally located within the flap.

**Figure 4:**
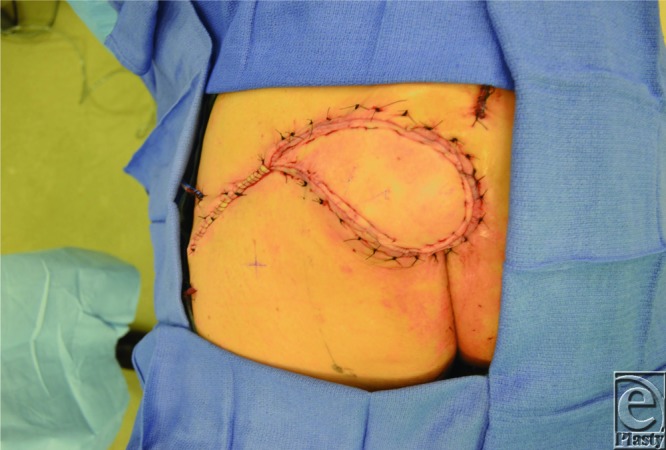
Immediate postoperative result following flap inset. The medial aspect of the flap was de-epithelialized and placed within the deep aspect of the defect to obliterate dead space.

The SGAP flap is perfused by perforators that arise from the superior gluteal artery (SGA), which emerges superior to the piriformis muscle. When designing an SGAP flap, a line is first drawn from the posterior superior iliac spine to the greater trochanter, since the SGA and its perforators are typically located along the medial two thirds of this line.^3^ A hand-held Doppler probe is then used to identify individual perforators, around which the skin paddle is designed (Fig 2). Operatively, the skin is incised and dissection carried down to the subfascial layer, after which suprafascial dissection is performed to identify perforators. Then, intramuscular dissection through the gluteus maximus muscle is performed (Fig 3) to gain vessel length to facilitate transposition. The SGAP flap can be delivered to the defect via rotation or V-Y advancement (Fig 4).^4^

The SGAP flap represents an important option for perineal reconstruction. It provides a large amount of well-vascularized tissue, including when dead space obliteration is needed. In addition, as a perforator flap that does not require any muscle sacrifice and where the donor site can be closed primarily, it involves minimal donor site morbidity. Finally, in patients with cancer, it is typically outside the zone of prior resection and radiation therapy.

## References

[1] Copeland LJ, Hancock KC, Gershenson DM, Stringer CA, Atkinson EN, Edwards CL (1989). Gracilis myocutaneous vaginal reconstruction concurrent with total pelvic exenteration. Am J Obstet Gynecol.

[2] Lacey CG, Stern JL, Feigenbaum S, Hill EC, Braga CA (1988). Vaginal reconstruction after exenteration with use of gracilis myocutaneous flaps: the University of California, San Francisco experience. Am J Obstet Gynecol.

[3] Cheon YW, Lee MC, Kim YS, Rah DK, Lee WJ (2010). Gluteal artery perforator flap: a viable alternative for sacral radiation ulcer and osteoradionecrosis. J Plast Reconstr Aesthet Surg.

[4] Chao AH, McCann GA, Fowler JM (2014). Alternatives to commonly used pelvic reconstruction procedures in gynecologic oncology. Gynecol Oncol.

